# Quantitative Phase Imaging Detecting the Hypoxia-Induced Patterns in Healthy and Neoplastic Human Colonic Epithelial Cells

**DOI:** 10.3390/cells11223599

**Published:** 2022-11-14

**Authors:** Igor Buzalewicz, Monika Mrozowska, Alicja Kmiecik, Michał Kulus, Katarzyna Haczkiewicz-Leśniak, Piotr Dzięgiel, Marzenna Podhorska-Okołów, Łukasz Zadka

**Affiliations:** 1 Bio-Optics Group, Department of Biomedical Engineering, Faculty of Fundamental Problems of Technology, Wroclaw University of Science and Technology, 50-370 Wroclaw, Poland; 2 Division of Histology and Embryology, Department of Human Morphology and Embryology, Wroclaw Medical University, 50-368 Wroclaw, Poland; 3 Division of Ultrastructural Research, Wroclaw Medical University, 50-368 Wroclaw, Poland

**Keywords:** colorectal cancer, hypoxia, digital holotomography, phenotyping, CRC, DHT

## Abstract

Hypoxia is a frequent phenomenon during carcinogenesis and may lead to functional and structural changes in proliferating cancer cells. Colorectal cancer (CRC) is one of the most common neoplasms in which hypoxia is associated with progression. The aim of this study was to assess the optical parameters and microanatomy of CRC and the normal intestinal epithelium cells using the digital holotomography (DHT) method. The examination was conducted on cancer (HT-29, LoVo) and normal colonic cells (CCD-18Co) cultured in normoxic and hypoxic environments. The assessment included optical parameters such as the refractive index (RI) and dry mass as well as the morphological features. Hypoxia decreased the RI in all cells as well as in their cytoplasm, nucleus, and nucleoli. The opposite tendency was noted for spheroid-vesicular structures, where the RI was higher for the hypoxic state. The total volume of hypoxic CCD-18Co and LoVo cells was decreased, while an increase in this parameter was observed for HT-29 cells. Hypoxia increased the radius and cell volume, including the dry mass of the vesicular content. The changes in the optics and morphology of hypoxic cells may suggest the possibility of using DHT in the detection of circulating tumor cells (CTCs).

## 1. Introduction

The appropriate oxygen saturation is essential for the existence and proper functioning of all multicellular organisms, but extreme depletion levels of this chemical compound that occur during hypoxia and its excessive concentrations characteristic of a hyperoxic milieu can induce cellular stress and prevent the maintenance of adequate homeostasis. Consequently, cells were forced to develop mechanisms and induce specific phenomena to counteract the oxygen value shifts. To oppose the prolonged hypoxia effect, the cells worked out pathways resulting in metabolic modifications such as downregulation of oxidative phosphorylation, inhibition of fatty acid desaturation, or altered expression of hypoxia-sensitive transcription factors [1,2,3]. This adaptability is a common event not only for normal epithelium but also for cancer cells. In particular, hypoxia in solid tumors plays a significant role in cancer progression, metastasis, and the effectiveness of implemented therapy. In the case of tumor cluster cells, the hypoxic regions are a consequence of abnormalities in the vascular beds, which supply oxygen and nutrients to an intensively growing tumor nest. Hypoxia can affect cancer-specific pathways leading to genomic instability and causing mutations; lead to a shift to aerobic glycolysis; affect the regulation of angiogenesis and cellular proliferation; lead to limitless replicative potential, apoptosis, and immune evasion; or promote the formation of distant metastases, which as consequence lead to worsening the prognosis for patient recovery [4]. Therefore, numerous studies are currently being conducted to characterize the changes that occur under hypoxia in cancer cells of different tumor subtypes [5,6,7,8,9,10,11,12,13]. Moreover, it should be noted that the existence of oxygen depletion enables the antitumor therapy focused on activated prodrugs and gene therapy targeting the hypoxia-inducible factor 1 transcription factor and recombinant anaerobic bacteria [2,14,15]. Thus, the accurate and detailed characterization of hypoxia-induced changes that occur at the cellular level is of significant importance.

A challenge here is also the use of a suitable technique that allows a highly sensitive, comprehensive quantitative and qualitative investigation of changes at the level of tissue and cellular structures. Among hypoxia imaging techniques such as positron emission tomography (PET), single photon emission computed tomography (SPECT), electron paramagnetic resonance (EPR), and magnetic resonance imaging (MRI), optical techniques (bioluminescence and fluorescence) still play a major role in the examination of hypoxia at the cell and tissue level [16,17]. The hypoxic environment may also be detected using hyperspectral imaging supported by fluorescent/phosphorescent boron nanoparticles [18], specific near-infrared optical imaging probes [19], two-photon fluorescence microscopy [20,21,22], and fluorescence resonance energy transfer (FRET) [23,24], which were implemented to monitor hypoxia in solid tumors or living cells. However, it should be noted that the main disadvantages of fluorescence imaging related to the use of immunofluorescence for staining are associated with permanent modification of the sample, photobleaching, and phototoxicity or interference caused by other target proteins.

In this study, we demonstrate the use of digital holographic tomography (DHT) based on quantitative phase imaging [25,26] to examine hypoxia-induced morphological, structural, and biochemical changes of colorectal cancer and healthy colonic epithelium cells. DHT is a non-destructive, label-free technique, in which, contrary to conventional microscopic imaging techniques, the refractive index (RI) is used as intrinsic contrast imaging rather than light intensity [27]. It not only allows the qualitative assessment characteristic for classical bright-field, phase-contrast, differential interference contrast, or fluorescence microscopic techniques, but it is also is much more accurate by providing additional quantitative information. The RI is a physical parameter directly related to the density and chemical content of the object examined [28,29]. Therefore, DHT providing RI data in the form of a three-dimensional (3D) refractive index distribution (3D-RI tomograms) simplifies not only the characterization of the morphometric properties of examined objects such as cell volume or size but also biophysical properties as chemical component concentration, dry mass density, or dry mass [30,31]. Current scientific findings indicate that these advantages make DHT a suitable technique for the complex phenotyping of biological objects such as tissues; single cells (eukaryotic/prokaryotic); and intracellular organelles or structures such as cytosol, nucleus, nucleoli, mitochondria, lipid droplets, and extracellular vesicles [32,33,34,35,36,37].

In a recent study, the DHT method was used for the comparison of hypoxia stress-induced changes in the normal colon epithelium as well as human adenocarcinoma cells. To our knowledge, this is the first attempt to use the DHT technique for the characterization of the stress-induced effect of the low-oxygen cellular environment. Significant changes in RI values between the normal colonic epithelium and colorectal cancer cells, including their microanatomical structures (cytoplasm, nucleus/nucleoli, vesicles), were determined. The total volume, dry mass, and size distribution of intracellular vesicles were also assessed. The ultrastructural architecture of the cell cultures used in this study was independently examined by transmission electron microscopy (TEM). Verification of hypoxic conditions was performed using confocal microscopy. The proposed approach provides novel perspectives in the quantitative rather than qualitative label-free optical phenotyping of single cells under hypoxic conditions based on RI data for digital pathology.

## 2. Materials and Methods

### 2.1. Cell Culture and Seeding

The human colorectal cancer cell lines HT-29 (grade 1) and LoVo (grade 4) as well as CCD-18Co cells representing the normal colonic epithelium were obtained from ATCC (Manassas, VA, USA). HT-29 cells were cultured with McCoy’s 5A (ATCC), LoVo cells with F12-K media (ATCC), CCD18-Co with Eagle’s minimum essential medium (Lonza, Basel, Switzerland) with 1% sodium pyruvate and 1% nonessential amino acids (all from Sigma-Aldrich, St. Louis, MO, USA). All media contained 1% l-glutamine and penicillin-streptomycin solution and 10% fetal bovine serum (Sigma-Aldrich). Cell cultures were provided in 5% CO 2 at 37 °C and 95% humidity. The medium was changed twice a week, and cells were passaged at approximately 70% confluence and trypsinized with 0.25% trypsin–EDTA solution (Sigma-Aldrich).

### 2.2. Induction of Hypoxic Stress, Cell Fixation, and Confocal Imaging

**Figure 1:**
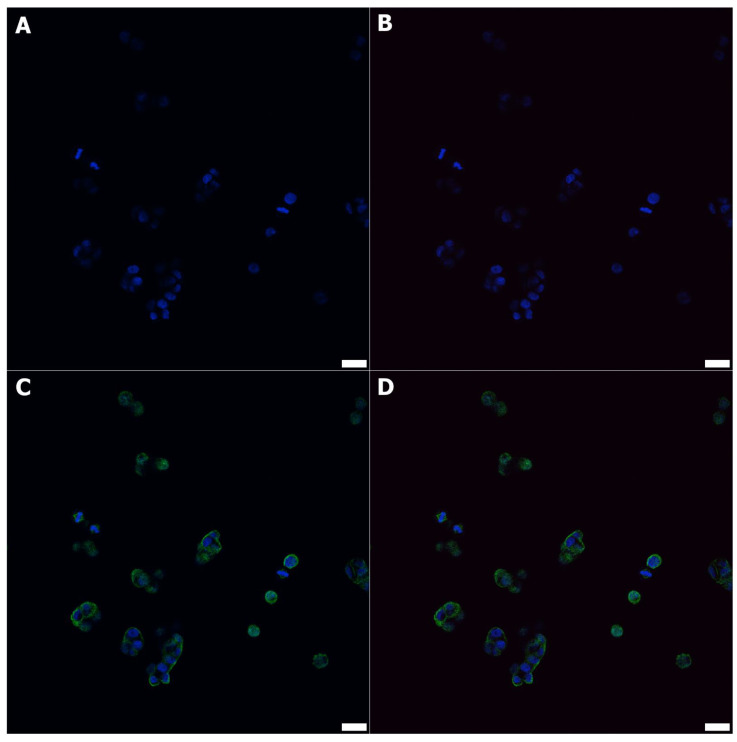
The immunofluorescence labeling against nuclei (blue DAPI), HIF1α (red), and β-actin (green) in normoxic HT-29 cells (**A**–**D**) showed no HIF1α-positive cells (**B**,**D**). All cells were positive on DAPI (**A**–**D**) and β-actin (**C**,**D**); scale bars = 20 μm.

**Figure 2:**
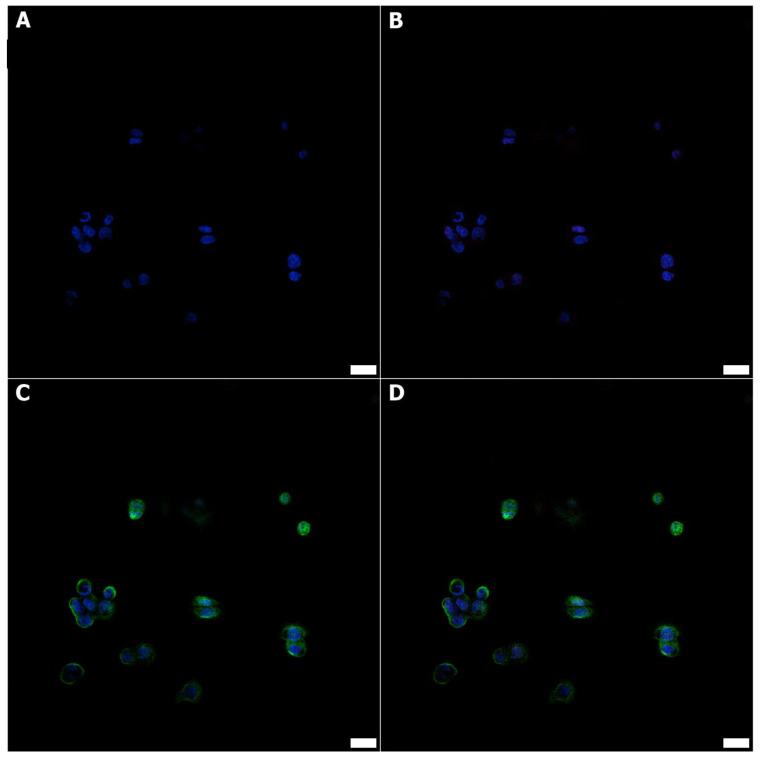
The immunofluorescence labeling against HIF1α (red) showed positive nuclear expression in hypoxic HT-29 cells (**B**,**D**). All hypoxic cells were positive on β-actin (green, (**C**,**D**)) and DAPI (blue nuclei, (**A**–**D**)); scale bars = 20 μm.

For each microculture, 600 µL of 2 × 10^4^ cells/mL was set up on Millicell EZ 8-well glass slides (Merck) and cultured for 24 h under hypoxia (1% O_2_) or normoxia conditions. After the incubation, the cells were fixed using 4% paraformaldehyde (PFA). Although the dehydrating tissue fixation methods may affect organelles’ structural integrity and their RI and reconstructed 3D-RI tomograms in DHT, the use of PFA enables the structural preservation of subcellular details; however, there is still a risk of changing their RI values [38]. Considering the above-mentioned advantages and limitations of the method, it was decided to choose PFA for cell fixation in order to avoid their exposure to oxygenation during transport. To avoid unwanted variations in the RI values, both cell cultures representing aerobic and hypoxic environments were fixed using an exact solution of PFA. Then, some of the samples were directly destinated for DHT imaging and the others were intended for immunofluorescence and TEM imaging. For the confocal microscopy, the slices were incubated at 4 °C overnight with primary specific polyclonal rabbit anti-HIF1α (1:400 dilution; Cell Signaling, Danvers, MA, USA) and mouse monoclonal anti-β-actin antibodies (1:100 dilution Cell Signaling, Danvers, MA, USA). Next, the slides were incubated for 1 h with donkey anti-rabbit secondary Alexa Fluor 568 conjugated antibody (1:2000 dilution; clone, Invitrogen, Carlsbad, CA, USA) and donkey anti-rabbit secondary Alexa Fluor 488 conjugated antibody (1:2000 dilution; clone, Invitrogen, Carlsbad, CA, USA) at 4 °C overnight. After that time the preparations were mounted using the Prolong DAPI Mounting Medium (Invitrogen). The observations were made at objective 60×/1.40 oil using Fluoview FV3000 confocal microscopy (RRID:SCR_017015, Olympus, Tokyo, Japan) coupled with Cell Sense software (RRID:SCR_016238, Olympus, Tokyo, Japan). The representative immunofluorescence staining performed on normoxic (Figure 1) and hypoxic HT-29 cells (Figure 2) is shown.

### 2.3. DHT Imaging of the Single Cells

**Figure 3:**
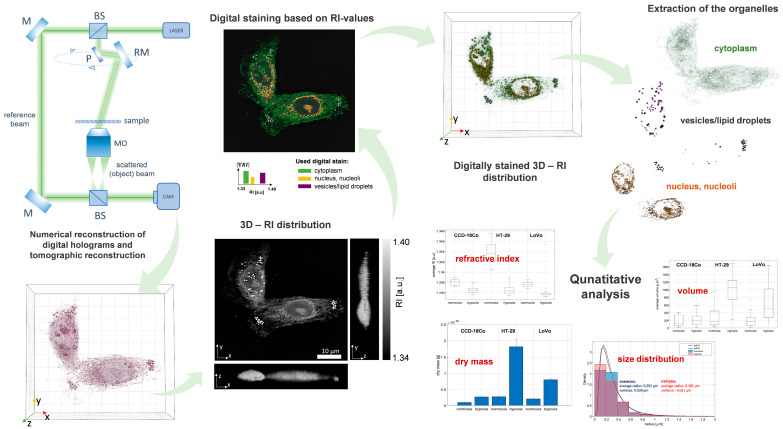
The schema of the DHT system (BS—beam splitters, M—mirror, RM—rotating mirror, P—prism, MO—microscopic objective) and the proposed RI data analysis algorithm.

Holotomographic imaging was performed on 3D Cell Explorer (Nanolive, Tolochenaz, Switzerland). In general, DHT as a quantitative phase imaging technique is based on the principles of digital holographic microscopy and optical diffraction tomography. Therefore, the used DHT system was based on a commercial off-axis Mach-Zehnder interferometric setup with a rotating mirror arm (see Figure 3). The object beam in a form of light scattered by the sample was transmitted by the microscopic objective to the camera. The camera recorded the series of digital holograms resulting from the interference of the plane reference beam and the object beam for different angles of sample illumination. The phase and amplitude of the object beam were numerically retrieved from the series of recorded digital holograms and then processed to reconstruct the 3D RI distributions. The used tomographic reconstruction algorithm was based on the Fourier diffraction theorem within the first-order Rytow approximation. Detailed descriptions of the holotomographic data processing implemented in the used commercial software STEVE (Nanolive, Tolochenaz, Switzerland), which was applied for the determination of the 3D RI, can be found in [26]. The field of view of the used DHT system was equal to 90 × 90 × 30 µm. The lateral and axial resolutions were equal to 190 nm and 400 nm, respectively.

The initially fixated CCD-18Co, HT-29, and LoVo in the number of 105 cells were placed in a cell culture µ-Dish (35 mm low, Ibidi, Gräfelfing, Germany). For each sample type (CCD-18Co, HT-29, LoVo), cell cultures from the normoxia and hypoxia groups were cultured in the same way. Therefore, the only extrinsic factor affecting the cells and altering the distribution of the RI was the oxygen concentration. For each sample of each type of examined cells (CCD-18Co, HT-29, LoVo), 15–17 3D-RI tomograms, each containing the 95 slices (2D-RI tomograms), were reconstructed. In total, 95 3D-RI tomograms and 9120 2D-RI tomograms were analyzed. The measurements of the RI value of the mounting medium were performed using the Abbe refractometer (NAR-2T, minimum scale: 0.001, ATAGO Co. Ltd., Tokyo, Japan) at 20 °C. Its RI value was equal to 1.334 and was used during the numerical reconstruction of the digital holograms.

### 2.4. Quantitative Analysis of Hypoxia-Induced Changes of Colorectal Cancer Cells

The use of spatial distributions of the refractive index makes it possible not only to determine local changes in the density and chemical composition of whole cells and their internal structures caused by physiological and external factor-induced processes, but also to analyze related morphological abnormalities. Therefore, the RI data and additional parameters extracted from them were used for phenotyping of the hypoxia stress among the fixed normal colon epithelium and adenocarcinoma cells (see Figure 3).

### 2.5. Analysis of the Influence of Hypoxic Stress on Refractive Index of Single Cells and Their Organelles

First, the variation of the average RI values of whole cells and changes in their morphology related to hypoxic stress were determined. The reconstructed 3D-RI tomograms of examined cells were denoised by the RI threshold approach to improve the contrast, limit the experimental noise, and accomplish cell segmentation. Based on the integration of the voxels of 3D-RI tomograms of cells, the total values of single-cell volume were determined. Then they were transformed into 2D-RI tomograms using maximum intensity projections to determine the ranges of RI values related to each cell line, which were used for digital staining of whole cells in STEVE software to obtain 3D rendered visualizations of these cells.

It was assumed that hypoxia-induced changes in the average RI values of the whole cell are directly related to changes in the RI and density of the constituent structures or organelles of the cell. Therefore, studying changes in the RI of specific cellular organelles can extend our knowledge of changes in the chemistry and density of these structures directly related to hypoxic stress. In our study, the following cellular structures were characterized, such as the cytoplasm, nucleus/nucleoli, and spheroid-shaped structures (lipid vesicles/droplets). Based on the 3D-RI tomograms, after RI-threshold-based segmentation of these structures, the ranges of RI values that directly related to them were determined and used for digital staining to obtain the 3D rendered visualizations.

### 2.6. Analysis of the Influence of Hypoxic Stress on Vesicles/Lipid Droplet-like Structures

To more precisely examine the influence of hypoxia on the vesicles/lipid droplets, their additional characteristics from the RI data such as total volume, dry mass, size distribution, and mean radius were calculated. First, the voxels with RI values higher than the threshold RI value were segmented to directly indicate the voxels related to the structures analyzed. By integrating these voxels, the volume of vesicles/lipid droplets in each sample was determined. After that, it was possible to calculate the dry mass of these structures. It was determined based on the linear calibration model [30,39] with the median value of the average RI of segmented spheroid-shaped vesicles/lipid droplets and their average volume. Since it was not possible to directly indicate that the observable spheroid-shaped structures were vesicles or lipid droplets, in this model, it was assumed that the refractive index increment is equal to 0.160 mL/g and that they contain proteins and lipids. Next, to analyze changes in the size distribution of these structures inside the cell under hypoxia stress, RI data processing from our previous work was applied [35]. The series of 2D-RI tomograms were digitally stained based on the determined RI values to indicate the spatial location of vesicles/lipid droplets, and after contrast enhancement, they were converted to binary masks. Next, based on the initial diameter evaluation, the range of the vesicles, and the determination of the circularity parameter, the regions of the vesicular objects were automatically extracted, and their size was determined. Subsequently, histograms representing the distribution of the number with a specific radius were prepared. For each histogram, the probability density function based on the Birnbaum–Saunders distribution with support at (0, ∞) exhibiting the lowest variation of radius of these structures was fitted. All 2D-RI tomogram processing was performed in MATLAB R2021b (MathWorks Inc., Natick, MA, USA), except the extraction of the vesicular regions, automatic counting, and determination of their size performed in freeware ImageJ software [40].

### 2.7. Transmission Electron Microscopy (TEM)

The CCD-18Co and human colorectal adenocarcinoma cells were fixed at RT in the solution of 3.6% (*v*/*v*) glutaraldehyde and cacodylate buffer (0.1 M, pH 7.4, SERVA Electrophoresis, Heidelberg, Germany) with saccharose (Chempur, Piekary Śląskie, Poland). The fixative was leached four times with the cacodylate buffer in the next step. Subsequently, each cell line was harbored inside a clot derived from bovine thrombin (Biomed, Lublin, Poland) and fibrinogen (3 mg/mL; Merck KGaA, Darmstadt, Germany). Then, the samples underwent fixation (1 h, RT) for the second time in 1% (*w*/*v*) osmium tetroxide OsO4 (SERVA Electrophoresis) diluted in 0.1 M cacodylate buffer to improve the contrast of the cell membranes and preserve the cytoplasmatic lipids. After washing away the OsO4 with the cacodylate buffer, dehydration of the specimens was conducted in increasing concentrations of ethanol (30–90%, Stanlab, Lublin, Poland). A mixture of 90% ethanol:90% acetone and a graded acetone series (90%, 95%, and 100%; Stanlab, Lublin, Poland) totally replaced the water in the samples. Afterward, the cells were infiltrated first in increasing ratios of acetone and Epon 812 (SERVA Electrophoresis): 3:1 (20 min), 1:1 (60 min), and 1:3 (60 min). The following day, the material was placed in an oven heated to 60 °C in rubber molds (Pelco, Ted Pella, Redding, CA, USA) filled with resin and 2,4,6-tris (dimethylaminomethyl) phenol as polymerization accelerator. After 7 days, the Epon blocks were sectioned with a Power Tome XL ultramicrotome (RMC, Tucson, AZ, USA) and diamond knife (Diatome, Nidau, Switzerland). Semithin 600 nm thick sections were stained with toluidine blue (SERVA Electrophoresis). The ultrathin sections mounted onto rhodium–copper grids (Maxta form, Ted Pella, Redding, CA, USA) were floated onto droplets of uranyLess solution and Reynold’s lead citrate 3% (Electron Microscopy Sciences, Hatfield, PA, USA) at RT for 1 min per each fixation. The last step was washing the grids 5 times in beakers filled with demineralized water and examining them using a JEM-1011 transmission electron microscope (JEOL, Tokyo, Japan) working at 80 kV accelerating voltage. The TEM imaging platform iTEM1233 fitted with a Morada camera (Olympus, Münster, Germany) allowed for capturing of electron micrographs.

### 2.8. Measurements of Ultrastructural Cell Traits

TEM images were analyzed with the freeware GNU Image Manipulation Program (GIMP ver. 2.10.8). The percentage of visible cell surface occupied by the lipid droplets was calculated as the pixel count of manually selected lipid droplets divided by the pixel count of the whole cell. Cell length was evaluated with a measurement tool. Autophagic vacuoles and autophagolysosomes were manually counted.

## 3. Results

### 3.1. DHT-Based Qualitative and Quantitative Analysis of the Colonic Cells under Hypoxic Conditions

**Figure 4:**
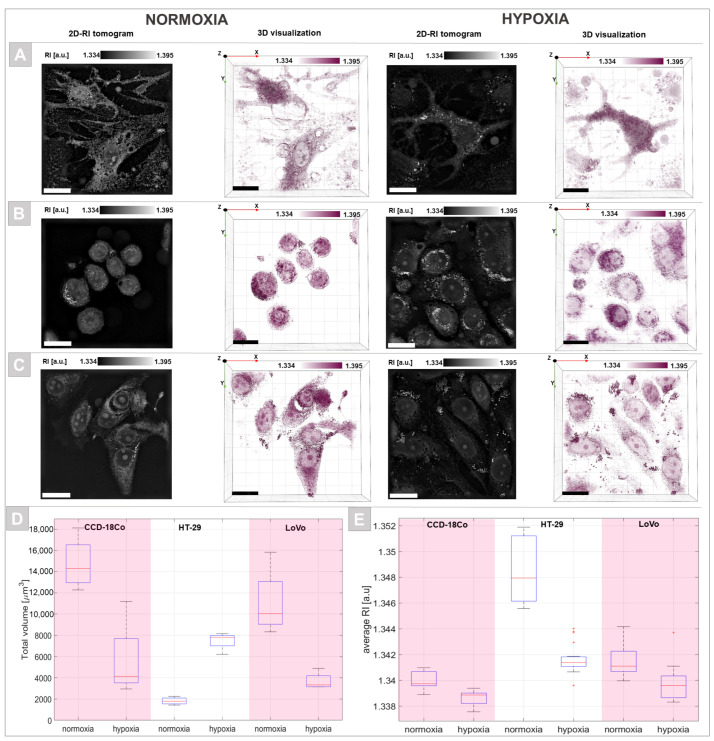
A comparison of the representative 2D-RI tomograms and 3D visualizations (scale bars: 20 µm) of CCD-18Co (**A**), HT-29 (**B**), and LoVo (**C**) cells under normoxic and hypoxic conditions. The variation of single-cell total volume (**D**) and average RI value of cells (**E**) was also considered.

The obtained RI data providing direct quantitative and qualitative information about the examined single cells were used for the characterization of the hypoxia-induced features in selected cell cultures. These results are shown in Figure 4. The 2D-RI tomograms related to the cross sections of the examined cells as well as digitally stained 3D-RI tomograms (Figure 4A–C) enabled the visualization of particular intracellular structures including cytoplasm, nucleus, nucleoli, and vesicles/lipid droplet-like structures due to the contrast variation directly correlated with the differences in their chemical composition and density. Induced hypoxic stress caused a significant change in single-cell morphology. Cells that were cultured in the hypoxic conditions had a more elongated shape similar to that of fibroblast cells, suggesting the potential rearrangement of the cytoskeleton. In the case of CDD-18Co and LoVo cells, hypoxia leads to the lengthening of these cells and decreases their volume. However, in the case of HT-29 cells, which initially had a spheroid-like shape in normoxia, hypoxic stress induced an increase in cell area. In the case of the single-cell volume change shown in a hypoxic environment (Figure 4D), a similar effect was observed. The decrease in cell volume of CCD-18Co and LoVo cells in a hypoxic milieu was associated with the opposite tendency that occurred in HT-29 cells. Quantitative analysis of the variation of the average RI of whole cells (Figure 4E) indicated that adenocarcinoma cells (HT-29, LoVo) had significantly higher RI values than normal epithelial cells, particularly HT-29 cells, correlating with our previous results [35]. However, hypoxia induced a statistically significant decrease in the RI values of the observed cells, suggesting that local changes occur in the density and chemical composition of the cellular content.

### 3.2. DHT-Based Quantitative Analysis of the Hypoxic Colonic Cells’ Structures

**Figure 5:**
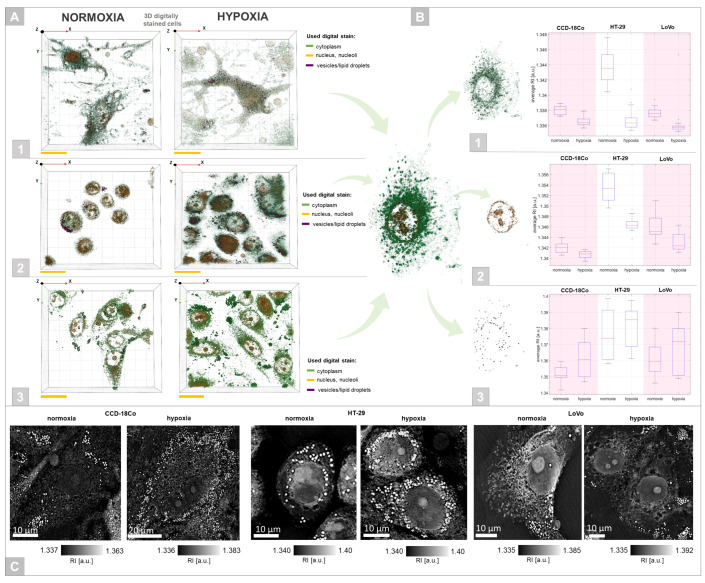
(**A**) The digitally stained 3D visualizations of CCD-18Co (1), HT-29 (2), and LoVo (3) cells under normoxic and hypoxic conditions (scale bars: 20 µm). (**B**) The boxplots representing the variation of the average RI values of cytoplasm (1), nucleus and nucleoli (2), and vesicular content (3) and their digitally stained representative visualizations. (**C**) The representative 2D-RI tomograms indicating the location of vesicles/lipid droplet-like structures inside the single-cells.

This analysis was focused on the extraction of quantitative information and biophysical parameters from reconstructed 3D-RI data. Representative results are shown in Figure 5. The specific RI values of individual intracellular structures related to their density and chemical composition enable the visualization of these structures in a truly label-free manner by digital staining based only on their RI values (Figure 5A). Quantitative analysis was particularly focused on intracellular structures such as cytoplasm, nucleus/nucleoli, and spheroid-shaped vesicles/lipid droplet-like structures to determine the hypoxia-induced changes in the properties of these organelles. Moreover, it was possible to quantitatively analyze the changes in these structures. Furthermore, it was shown that there exist significant differences between the RI values of these cellular compounds under normoxic and hypoxic conditions (Figure 5B). In the case of the cytoplasm and nucleus/nucleoli, the RI decreased with the limitation of oxygen concentration, which indicated the changes in the chemical composition of these structures, which were less dense under hypoxia.

**Figure 6:**
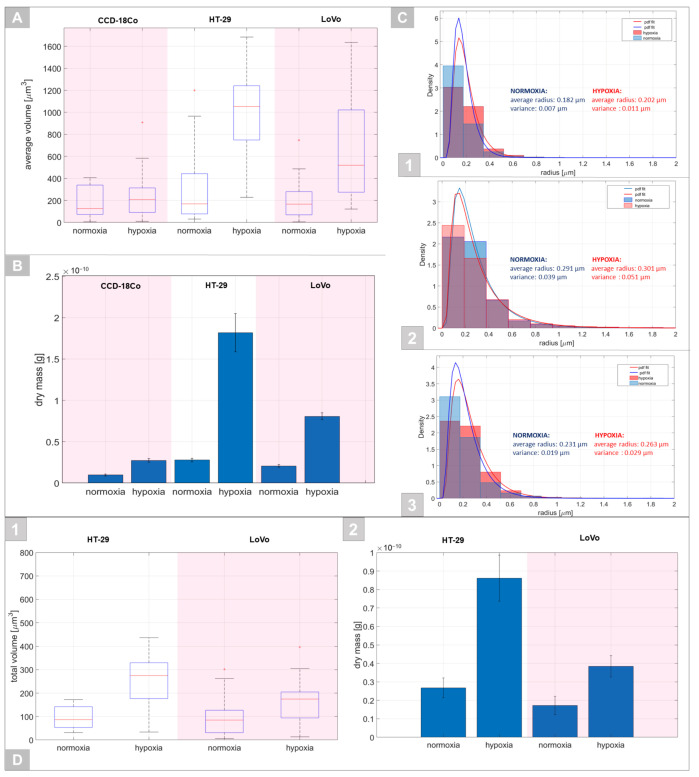
(**A**) The variation of the average volume of the vesicles/lipid droplet-like structures of examined cells in both normoxic and hypoxic conditions. (**B**) A comparison of the dry mass of detected structures in analyzed samples under normoxic and hypoxic conditions. (**C**) The determined distribution of the radius of these structures in analyzed cultures: CCD-18Co (1), HT-29 (2), LoVo (3) cells under normoxic and hypoxic environments with fitted probability density functions (pdf). (**D**) The comparison of the variation of the total volume of the high RI fraction (RI > 1.375) structures in cancer cells (1) and their dry mass (2).

The changes occurred in the case of all cells examined; however, they were more significant in the case of cancer cells, particularly for HT-29 cells. A different tendency occurred for vesicular content and lipid droplet-like structures, which exhibited the highest average RI among all analyzed organelles (Figure 5C). It was not possible to directly indicate whether these structures corresponded to vesicles or lipid droplets; however, hypoxic stress induced an increase in their RI values no matter which cell line was analyzed. The highest RI was also obtained for cancer cells, particularly HT-29. Visual analysis of the RI tomograms showed that under normoxia and hypoxia, there were significant differences between the number and size of these vesicles/lipid droplet-like structures among cells. Therefore, an additional examination of their volume inside the cells, size distribution, and dry mass was performed (Figure 6).

It was shown that for CCD-18Co cells, the average volume of these structures within the cells was not changing noticeably under hypoxia (Figure 6A). However, hypoxic cells of HT-29 and LoVo showed a significant increase in their average volume, which indicates that the concentration of these structures may be directly related to hypoxia. Furthermore, the determined dry mass of detected cellular objects was increased under hypoxic conditions (Figure 6B), suggesting that their cargo was denser. A comparison of the size distribution of these spheroid-shaped structures changed when the oxygen concentration decreased (Figure 6C). Generally, under hypoxia, the average radius of detected structures was increased and showed the highest values for cancer cells, particularly for HT-29 cells. In the case of the healthy colonic cells, under normoxia, a higher number of these structures had a radius below 185.7 nm, while under hypoxia, the examined objects showed higher values of the radius. In particular, the number of structures with radii from 185.7 nm to 371.4 and 371.4 nm to 557.1 nm was increased under hypoxia. A similar tendency was observed in the case of the LoVo cells. However, the number of structures with a radius in the range of 185.7 to 371.4 nm was lower and distributed in the range of 371.4–557.1 nm, which was higher than that in the case of CCD-18Co cells. The opposite tendency occurred in the case of HT-29 cells. These cells demonstrated that the highest fraction of detected structures had a radius value below 187.1 nm under hypoxia and between 187.1 and 371.4 nm under normoxia.

For comparison of the possible concentration differences of lipid droplets in the cancer cells under hypoxic/normoxic conditions, high-RI spheroid-shaped structures (RI > 1.375) were extracted from 3D-RI tomograms, and their total volume and dry mass were determined (Figure 6D). It can be seen that a greater distribution of lipid droplets under hypoxia was observed in the case of the HT-29 cell line than in LoVo cells. Furthermore, the dry mass of the lipids in HT-29 was as twice high as that in LoVo cells.

### 3.3. TEM Imaging Qualitative and Quantitative Analysis

**Figure 7:**
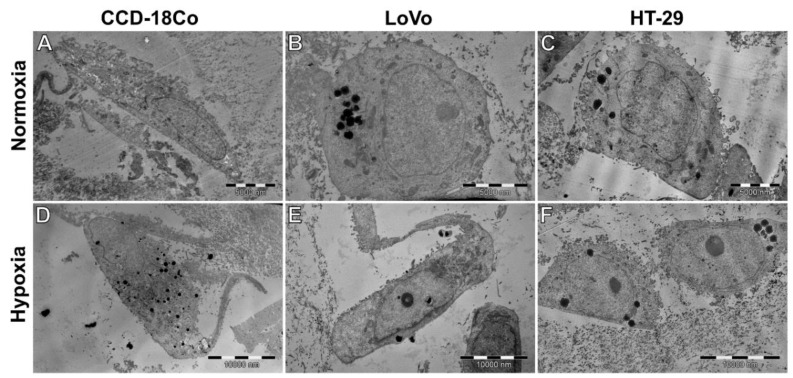
A comparison of cell morphology in normoxia (**A**–**C**) and hypoxia (**D**–**F**) in examined cell lines. No major differences were visible, besides elongated protrusions in hypoxic CCD-18Co cells (**D**). Regardless of oxygenation, cells tend to exhibit visible exocytic activity; they maintain similar shape and morphology of nuclei.

For TEM analysis, 1.5 × 10^6^ cells per 35 mm µ-Dish of each cell line were used as specimens. Qualitative cell visualization by TEM revealed a more elongated shape of the cells under hypoxic conditions, which was a particularly characteristic phenomenon in the case of CCD-18Co cells (Figure 7A,D).

**Figure 8:**
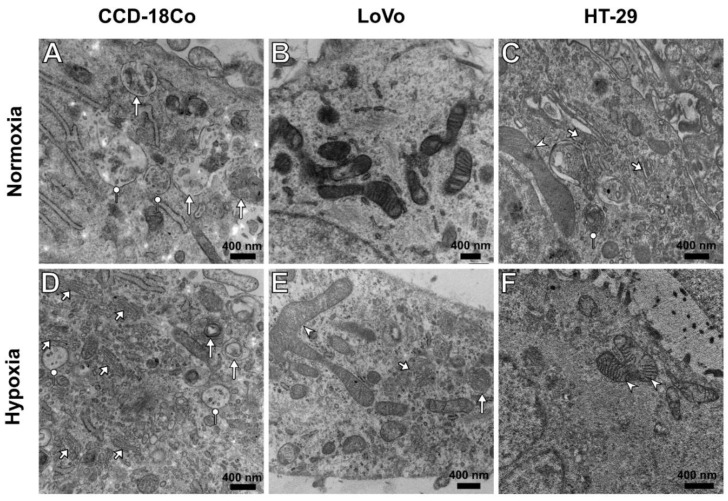
Micrographs of representative cells’ cytoplasm in normoxia (**A**–**C**) and hypoxia (**D**–**F**). (**A**,**D**) CCD-18Co cells in lower oxygenation tend to have more autophagic vacuoles (arrows) and multivesicular bodies (MVBs; dot marker), as well as more numerous Golgi apparatuses (short arrows). (**B**,**E**) Mitochondria in the LoVo cell line tend to be larger and fused (arrowhead), suggesting the dominance of fusion over the fission process. MVBs and autophagic vacuoles are less numerous than those in the CCD-18Co cell line. (**C**,**F**) Hypoxic mitochondria in HT-29 tend to be less electron-dense than the normoxic ones. No significant differences in autophagy or exocytosis were observed (n = 3 cells/each cell line).

The TEM micrographs also showed lipid droplets (Figure 7). The presence of multivesicular bodies (MVBs) containing exosomes and individual autophagic vacuoles was confirmed in the identified vesicular structures (Figure 8). Exocytic activity was present in all the cells examined, although the greatest distribution of MVBs and autophagic vacuoles was demonstrated in hypoxic CCD-18Co cells (Figure 8A,D). In these cells, an increase in the biogenesis of extracellular vesicles was accompanied by a more extensive Golgi apparatus (Figure 8D). Regarding LoVo cells, there were noticeable changes in mitochondrial morphology between different oxygenation states (Figure 8B,E). On the other hand, HT-29 cells showed a lower electron density of mitochondria compared to those organelles in the normoxic environment (Figure 8C). TEM quantitative assessment included the three most representative cells from each cell line under both normoxic and hypoxic conditions. The percentage of the area occupied by lipid droplets, the cell length measured in nanometers, and the total number of autophagic vacuoles were taken into account. The mean was then counted from each value. Regarding quantitative examination, no significant differences were found in the respective oxygenation states for individual cells; in this case, the differences observed between different cell lines seemed to be more pronounced (Figure 8). However, the observed results should be considered with great caution because of the small number of cells analyzed and the invasive protocol for the preparation of samples for TEM imaging. The exact numeric values are shown in Table 1.

## 4. Discussion

The detected changes can be directly related to the hypoxia-induced biochemical process and the rearrangement of the internal structure of examined cells. As was confirmed in the case of the fibroblasts, hypoxia changed the cell area, cell volume, cell adhesive properties, and cellular motility in L929 fibroblasts [41]. It was confirmed that despite changes in cell volume and adhesion, these effects could be related to the stabilization of HIF-1α influencing rearrangement of cytoplasmic β-actin and changes in L929 cell morphology and function. The elongation of cells as the result of hypoxia could suggest a densification of the cytoplasm and intracellular structures leading to an increase in cell density and the average RI values. However, the results obtained, particularly the observed decrease in the average RI values of single cells, suggest that cells are less dense under hypoxia and significant changes in the chemical composition of intracellular structures may take place. To verify this assumption, it was decided to directly analyze changes in the RI values of the main cellular structures such as the cytoplasm, nucleus/nucleoli, and vesicles/lipid droplet-like structures. The higher RI of the cargo of these vesicles/lipid droplet-like structures under hypoxia may be related to the aerobic glycolysis characteristic of these cells inducing the production of vital secondary metabolites for the generation of lipids, proteins, and nucleic acids; promoting the cancer cell proliferation is a key role for the aerobic glycolysis characteristic of cancer cells [42]. Our previous results concerning the DHT examination of the extracellular vesicles for single cells indicated that the increase in their RI value corresponds to the increase in the tumor grade (HT-29/G1, LoVo/G4) [35]. However, in a recent study, the changes in the RI of examined structures indicated the opposite tendency, which may be directly related to hypoxia-induced stress. In the context of already reported [33,43] results that confirm that spheroid-shaped subcellular structures with RI values of 1.375 or higher correspond to the lipid droplets labeled with fluorescent probes, our recent results (Figure 5C) suggested that in analyzed spheroid-shaped structures, in addition to the subfraction corresponding to the vesicles, there exists a subfraction of structures (with RI > 1.375) directly corresponding to the lipid droplets. Moreover, on the variation of average RI values of these structures, it can be concluded that the increase in their RI values under hypoxic conditions may be related to the more numerous distributions of lipid droplets within the cells. Furthermore, the highest RI values of these structures were observed for cancer cells, particularly HT-29 cells, rather than in CCD-18Co cells, which may also support our suggestions about the presence of lipid droplets. Their presence can be expected since they are a key organelle responsible for cancer cell survival under hypoxia due to their activities as scavengers of reactive oxygen species released from mitochondria in hypoxia-exposed cells [44]. Moreover, in contrast to normal cells, which mainly generate energy from adenosine triphosphate (ATP) by mitochondrial oxidative phosphorylation, cancer cells use glycolysis to generate ATP even under sufficiently oxygenated conditions (aerobic glycolysis or the Warburg effect) [45] accompanied by a higher concentration of lipid droplets. Furthermore, it was also indicated that the upregulated expression of hypoxia-inducible lipid droplets was common in the case of colonic cancers [46,47]. Obtained results have shown that generally, in the case of CCD-18Co and LoVo cells, these structures were located throughout the cell body, but predominantly in the peripherical regions of the cells (Figure 5C). In the case of HT-29 cells, these structures were generally located in the region surrounding the nucleus, which may be related to the presence of the endoplasmic reticulum (ER) in these regions, which is responsible for the synthesis of lipid droplets. Hypoxia facilitates fatty acid uptake into cancer cells, although this is a condition accompanied by suppression of de novo lipogenesis [3,47,48]. The increase in the lipid content inside the cells was correlated with the increase in the RI value and in consequence also the dry mass. The dry mass parameter was higher for cancer cells, which is expected in the case of nutrients such as glucose and glutamine, as neoplastic tissues exposed to hypoxia are insufficiently supplied with serum lipids and develop internal mechanisms to enhance lipid uptake in response to the hypoxic state. The hypoxia increased the dry mass of these structures inside the cancer cells, which may be related to the higher concentration of lipid components in these structures as lipid droplets. This is an important issue since cancer cells with increased concentrations of lipid droplets exhibit higher resistance to chemotherapy [49].

Moreover, it should be pointed out that during the examination of the influence of different external factors or induced stress (such as hypoxia) it is necessary to examine the changes in the average RI values of the organelles, rather than whole cells. In our case, hypoxia induced an increase in the average RI of the vesicles/lipid droplet-like structures in cancer cells, while the average RI values of whole cells were decreasing. This is caused by the fact that the total volume of the cytosol and nucleus/nucleoli inside the cell is significantly higher than the volume of the vesicles/lipid droplet-like structures. Therefore, the increase in the RI value of these structures is compensated by the decrease in RI of other analyzed organelles during the determination of the average RI of whole cells. Only the complex examination of the RI variation of specific organelles in our case enabled the discovery of the opposite tendency of RI changes of vesicles/lipid droplet-like structures in cancer cells.

## 5. Conclusions

The results presented in this work suggest the possibility of extending the currently available detection tools and methods towards early screening of solid tumors, especially in colorectal adenocarcinomas, by measuring the values of the above-mentioned optical and morphological parameters of the examined individual cells.

## 6. Perspectives

DHT may have the privileged potential to detect circulating tumor cells (CTCs) released from the primary tumor site and found in the systemic circulation as single cells or cancer cell clusters, which can be detected in a liquid biopsy [50]. Imaging by DHT is a quick and intuitive procedure, which with the relatively simple use of the device may bring new possibilities for the early detection of CTCs. The identification of CTCs is of increasing practical importance in today’s practical oncology [51].

One of the major difficulties in CTC examination is the small number of these cells that can be isolated from the blood. It is estimated that 10 mL of venous blood contains 0 to 10,000 CTCs [52]. An additional difficulty in patients with CRC is the fact that the total number of CTCs seems to be decreased compared to other solid cancers such as prostate or lung cancer [52]. This certainly has to do with the portal circulation, which first collects nutrient-rich venous blood from the intestines and then delivers it to the liver’s hepatocytes. The aforementioned anatomical condition is also related to the unfavorable phenomenon of CRC metastasis, where usually the liver, apart from regional lymph nodes, is the first site of forming distant metastases [53]. It is estimated that there are synchronous distant metastases at the time of CRC diagnosis in 25% of patients, while in the long term, autopsy studies have shown that as many as 75% of patients with CRC will develop metastases to the liver. The potential for metastasis formation in the liver is determined by the number of CTCs, which must be at least 10^6^ cancer cells present in the portal circulation to form a metastatic lesion [54]. These data suggest favorable implications for the use of CTCs as an independent marker of clinical staging and CRC progression. Despite the small number of CTCs in the blood, there is a confirmed relationship between the total number of CTCs and the tumor size as a strong positive correlation for both of these parameters [55]. Detection of CTCs using DHT may prove to be invaluable support in monitoring the progression of the disease and in determining its current clinical advancement, including response to the implemented treatment. According to estimates, one gram of neoplastic solid tissue releases from 10^5^ to 3 × 10^6^ CTCs per day, which enables the use of CTCs as a sensitive marker of residual disease [56] and in detecting possible recurrence of the neoplastic process. Current technological platforms used for isolation (capture and enrichment) and characterization are based on immunoaffinity and biophysical properties, which enable the CTC counting or molecular characterization by means of immunochemistry, RT-qPCR, fluorescence in situ hybridization (FISH), and DNA and RNA sequencing techniques. The other techniques used for direct analysis are based on molecular (RNA-based) assays [57]. The tests for the detection of CTCs, which have been available on the market for a long time, most often use the antigen–antibody reaction against markers of epithelial cells, such as the epithelial cell adhesion molecule (EpCAM), which is the most frequently used antigen for the detection of CTCs [52,56]. Popular immunocytochemical tests also use cytokeratins to detect neoplastic cells of epithelial origin [56]. However, it should be mentioned that the most biologically aggressive CTCs that show the ability to form new metastatic foci revealed intermediate biological features between the endothelial–mesenchymal transition (EMT) and the mesenchymal–epithelial transition (MET) phenotypes, and during the EMT process, some epithelial markers such as EpCAM are lost, which makes it impossible to detect CTCs using currently used immunocytochemical techniques or methods using antigen–antibody reactions against these biomarkers [52].

The main potential for the differentiation of CTCs by DHT is based on the spatially resolved RI data provided by this technique. The RI of single cells, being a key optical parameter, has already been shown to provide new insights into various biological systems at the cellular level and beyond, and it can be used to characterize and differentiate single cells, organelles, and various biophysical processes [58]. DHT, by providing a 3D-RI distribution of single cells, allows for their multiparametric characterization by RI values or volume, dry weight, and size distribution, making it potentially useful for disease diagnosis. However, it should be pointed out that these parameters can vary in range even for the same cell species. Therefore, RI data can provide meaningful and correlated information only for a large number of examined cells. Furthermore, the measurement of RI depends on chemical pretreatment (live or fixed samples) and extracellular conditions (chemical treatments, temperature, osmotic pressure, culture medium). Therefore, for any study aiming to correlate RI with cell status or disease diagnosis, standardization of culture conditions and measurements is necessary. Furthermore, the RI of cells exhibits low specificity for chemical molecules. However, it has great potential to complement existing biochemical techniques, such as fluorescent biomarkers, to provide more complete information. DHT can be readily integrated with fluorescence detection and, through the specificity provided by fluorescence biomarkers, can enable a more complex analysis of the cell’s refractive index in disease diagnosis.

**Figure 9:**
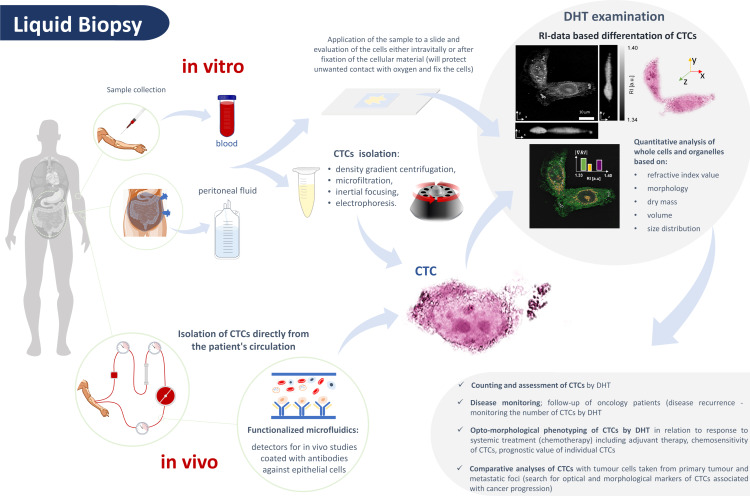
Possibilities of using the digital holotomography (DHT) method in the detection of circulating tumor cells (CTCs).

DHT enables the simultaneous assessment of the morphology of the tested cells and their optical parameters as well as immunofluorescence detection of selected markers, which is possible with the use of the latest devices available on the market. An advantage of DHT is the short analysis time and relatively low costs associated with the operation of the device—a specific financial outlay is required for the purchase of appropriate measuring equipment. The undoubted advantage of DHT is the possibility of performing additional analyses of single tumor cells that were previously examined on the glass slide using other DHT-supportive molecular techniques. Moreover, the specimens can be examined immediately after their collection at the patient’s bedside and do not require any special staining or additional isolation procedures. Verifying this hypothesis requires further research and multicenter randomized studies in this direction. The possibilities of the potential application of DHT in the detection and evaluation of CTCs are presented in Figure 9.

## Figures and Tables

**Table 1:** TEM quantitative assessment of morphometric and subcellular patterns of the human non-neoplastic and cancer cells of colonic epithelium appearing in different oxygenation conditions (n = 3 cells/oxygen environment in each line).

Cell Lines					
CCD-18Co		LoVo		HT-29	
Normoxia	Hypoxia	Normoxia	Hypoxia	Normoxia	Hypoxia
Means ± SDs of the percentage (%) of lipid droplet surface					
0	0.02	0.03	0.01	0.08	0.05
		±	±	±	±
		0.01	0.01	0.07	0.04
Means ± SDs of the autophagic vacuole number					
20 ± 5	14 ± 13.28	2 ± 1	4 ± 1.53	1 ± 1.73	1 ± 0.58
Means ± SDs of the cell length (nm)					
22,563.22	42,373.34	31,166.92	31,748.93	17,312.28	13,297.24
±	±	±	±	±	±
4763.64	6322.03	14,854.46	16,454.30	833.20	1763.62

TEM, transmission electron microscopy; CCD-18Co, healthy colonic epithelium; LoVo and HT-29, colonic adenocarcinomas.

## Data Availability

The data presented in this study are available on request from the corresponding author.
